# Effects of green tea extract treatment on erythropoiesis and iron parameters in iron-overloaded β-thalassemic mice

**DOI:** 10.3389/fphys.2022.1053060

**Published:** 2022-12-23

**Authors:** Kornvipa Settakorn, Sarawut Kongkarnka, Anchan Chompupoung, Saovaros Svasti, Suthat Fucharoen, John B. Porter, Somdet Srichairatanakool, Pimpisid Koonyosying

**Affiliations:** ^1^ Department of Biochemistry, Faculty of Medicine, Chiang Mai University, Chiang Mai, Thailand; ^2^ Department of Pathology, Faculty of Medicine, Chiang Mai University, Chiang Mai, Thailand; ^3^ Royal Project Foundation, Chiang Mai, Thailand; ^4^ Thalassemia Research Center, Institute of Molecular Biosciences, Mahidol University Salaya Campus, Nakorn Pathom, Thailand; ^5^ Red Cell Disorder Unit, Department of Haematology, University College London, London, United Kingdom

**Keywords:** green tea, EGCG, iron overload, thalassemia, erythropoiesis, erythropoietin, erythroferrone

## Abstract

β-Thalassemia is characterized by ineffective erythropoiesis leading to chronic anemia. Thus, increased iron absorption from the duodenum and via blood transfusions is required to maintain normal blood hemoglobin (Hb) levels and iron chelators in the removal of excessive iron. Certain agents are also needed for the improvement of stress erythropoiesis and iron dysregulation. Green tea extract (GTE), which is rich in epigallocatechin-3-gallate (EGCG), is known to possess radical scavenging and iron-chelating activities. We aimed to assess the effects of green tea extract on erythroid regulators, iron mobilization and anti–lipid peroxidation in the liver, spleen, and kidneys of iron-loaded β-globin gene knockout thalassemic (BKO) mice. Our results indicate that treatments of green tea extract and/or deferiprone (DFP) diminished levels of plasma erythropoietin (EPO) and erythroferrone (ERFE), and consistently suppressed kidney *Epo* and spleen *Erfe* mRNA expressions (*p* < .05) in iron- loaded BKO mice when compared with untreated mice. Coincidently, the treatments decreased plasma ferritin (Ft) levels, iron content levels in the liver (*p* < .05), spleen (*p* < .05), and kidney tissues of iron–loaded BKO mice. Furthermore, lipid-peroxidation products in the tissues and plasma were also decreased when compared with untreated mice. This is the first evidence of the orchestral role of green tea extract abundant with epigallocatechin-3-gallate in improving ineffective erythropoiesis, iron dysregulation and oxidative stress in iron-overloaded β-thalassemic mice.

## 1 Introduction

β−Thalassemia is a genetic disorder that is caused by ineffective β−globin chain synthesis of hemoglobin (Hb) (Cao and Galanello, 2010; Galanello and Origa, 2010; Origa, 2017). Impaired production of β−globin chains can lead to an excess of α−globin chains producing insoluble hemichromes in red blood cell (RBC) membranes (Yuan et al., 1992; Casu et al., 2020). Together with unstable Hb, these aggregates can damage the RBC membrane leading to hemolysis and chronic anemia (Jarolim et al., 1990; Grelloni et al., 1993). Accordingly, the ineffective erythropoiesis (IE) that is associated with chronic anemia can increase iron absorption *via* the duodenum, enhance systemic iron metabolism and accelerate erythropoietic activity in β−thalassemia patients (Ozturk et al., 2021). Under conditions of anemia and hypoxia, erythropoietin (EPO) production is markedly activated in the kidneys to compensate for RBC hemolysis leading to bone marrow expansion, skeletal changes, and hepatosplenomegaly (Rivella, 2012; Rivella, 2019). Coincidently, erythroferrone (ERFE) is synthesized by bone marrow and the spleen in response to EPO activation and regulation of iron metabolism, thereby inhibiting the production of hepcidin (master iron regulator) which is mainly synthesized in the liver (Kautz et al., 2014; Kautz et al., 2015; Rivella, 2019).

Furthermore, β−thalassemia patients can develop iron overload from multiple RBC transfusions, while β−globin gene knockout (BKO) thalassemic mice can develop iron overload by consuming a ferrocene-supplemented diet and through intraperitoneal injections of iron dextran (Koonyosying et al., 2018; Settapramote et al., 2021). As a consequence, excessive iron is accumulated in certain major organs including the liver, heart, macrophage, endocrine glands, and kidneys. Iron catalyzes the production of reactive oxygen species (ROS) through the Fenton reaction, which can then cause oxidative tissue damage (Dillard et al., 1984; Kang, 2001; Kohgo et al., 2008; Shander et al., 2009). Thus, standard iron chelators, such as desferrioxamine (DFO), deferiprone (DFP), and deferasirox (DFX) can be used to diminish iron accumulation in the patient’s body by interacting with redox labile iron pools and mobilizing ferritin iron; nonetheless, the employment of these chelators can have adverse effects (Viprakasit et al., 2009; Viprakasit and Origa, 2014; Kontoghiorghe and Kontoghiorghes, 2016; Mobarra et al., 2016).

Green tea extracts (GTEs) contain many polyphenolic catechins, of which epigallocatechin 3-gallate (EGCG) is the most abundant (Chacko et al., 2010; Khan and Mukhtar, 2013). Interestingly, EGCG manifests antioxidant, anticancer, radical−scavenging, iron−chelating and anti−inflammatory properties, and offers protective effects on iron−induced oxidative cardiac, kidney, liver, intestinal, and neuronal cells (Mandel et al., 2005; Avramovich-Tirosh et al., 2007; Kim et al., 2008; Bao et al., 2013; Al-Basher, 2019). Likewise, EGCG showed a protective effect against the damage of EPO−supplemented erythroid culture from X−ray irradiation (Monzen and Kashiwakura, 2012). Importantly, our previous studies have revealed that GTE, in particular EGCG, could effectively ameliorate and protect cells from iron overload-induced oxidative damage in BKO thalassemic mice and β−thalassemia patients (Srichairatanakool et al., 2006; Chansiw et al., 2021). Moreover, EGCG restored the reduced levels of glutathione(GSH) and inhibited apoptosis in 3−morpholinosydnonimine−induced fibroblasts in conjunction with glucose−6−phosphate dehydrogenase (G6PD) deficiency *via* the phosphatidylinositol−3−phosphate kinase/protein kinase B pathway, consequently thereby improving the survival of the cells (Ho et al., 2006). Furthermore, EGCG was found to inhibit the anion co−transporter on the plasma membrane and prevent the dehydration of sickle RBC induced by urea (Ohnishi et al., 2001). Inversely, GTE and standard EGCG showed a concentration−dependent pro−oxidant effect by depleting GSH content and increasing the amount of oxidized glutathione (GSSH) in G6PD deficient−RBC but not for their normal counterparts (Ko et al., 2006). Importantly, heavy consumption of tea containing high amounts of EGCG and tannins has been claimed to interfere with the duodenal absorption of dietary iron in both anemic and nonanemic people, possibly leading to iron-deficiency anemia (Kim et al., 2008; Fan, 2016; Lazrak et al., 2021). Recently, we have identified the binding of recombinant ERFE obtained from human embryonic kidney 293 cells to its receptor on the plasma membrane of human hepatocellular carcinoma (Huh7) cells, which could then suppress *Hamp1* gene expression (Than et al., 2021). Together, *ERFE* gene expression and ERFE production were increased by EPO stimulation, which would then act as a bone morphogenic protein (BMP) trap that diminishes hepcidin expression by sequestering BMP ligands away from their surface receptors on hepatocytes *via* the BMP/suppressor of mothers against the decapentaplegic (SMAD) signaling pathway (Arezes et al., 2018). Conversely, the nuclear factor erythroid 2−related factor 2 (Nrf2) was able to upregulate *Bmp6* expression and stimulate the Bmp6−hepcidin axis in hepatic sinusoid endothelial cells, thereby restoring BMP6 inhibition by ERFE in β−thalassemia mice (Lim et al., 2019). However, the association of green tea extract with erythropoietic activity has not yet been fully elucidated. This study examined the efficacy of GTE treatment on iron, oxidative stress, and erythropoiesis parameters in iron dextran−loaded BKO thalassemic mice.

## 2 Materials and methods

### 2.1 Chemicals and reagents

All organic solvents were of HPLC− or Analytical Reagent−grade and purchased from Fisher Scientific International Inc., Hampton NH, United States. Standard EGCG, catechin (C), epicatechin (EC), epicatechin−3−gallate (ECG) and epigallocatechin (EGC) (at least 95% pure) were purchased from Aktin Chemical Inc., Chendu, People’s Republic of China. Iron dextran was purchased from Santa Cruz Biotechnology, Santa Cruz, CA, United States. Ortho-phosphoric acid (H_3_PO_4_) and trichloroacetic acid (TCA) were purchased from Merck & Co., Inc., Keniworth, NJ, United States. Butylated hydroxytoluene (BHT), ferrous ammonium sulfate (FAS), 3−(2−pyridyl)−5,6−diphenyl−1,2,4−triazine−*p,p′*−disulfonic acid monosodium salt (ferrozine), sodium acetate, thiobarbituric acid (TBA), 1,1,3,3−tetramethoxypropane (TMP), thioglycolic acid (TGA), hematoxylin and eosin (H&E) dye solution, and Perl’s staining solution were purchased from Sigma−Aldrich Chemicals Company, St. Louis, MO, United States. Diethylpyrocarbonate (DEPC)−treated water, isopropanol and TRIzol were purchased from Thermo Fisher Scientific, Waltham, MA, United States. Sandwich ELISA kits for EPO (abx511437), ERFE (abx254979) and ferritin (abx195618) were purchased from Abbexa Company Limited, Milton, Cambridge, United Kingdom.

### 2.2 Preparation of green tea

GTE was prepared and used to determine EGCG content using the method previously established by Koonyosying and colleagues (Koonyosying et al., 2019). Fresh tea (*Camellia sinensis*) shoots were harvested from tea fields of the Royal Project Foundation in Chiang Mai and immediately inactivated with inherent polyphenol oxidase with the use of an electric microwave oven (800 W, 3 min, 100°C). Dry tea leaves were ground with an electric blender (SharpThai Company, Limited, Thailand), extracted with hot deionized water (DI) (80°C, 1 kg/10 L) for 10 min and filtered on a membrane (Whatman’s cellulose acetate type, 0.45 µm pore size, Sigma−Aldrich Chemicals Company, St. Louis, MO, United States) under a vacuum. The GTE filtrate was then lyophilized to the point of dryness and kept in a polyethylene bottle in the dark at -20°C until analysis. GTE was used to quantify EGCG using the HPLC system coupled with DAD (Model 1290 Infinity II) manufactured by Agilent Technologies, Inc., Santa Clara, CA, United States. The column was calibrated with authentic catechins including C, EC, ECG, EGC, and EGCG (1 mg/ml) and the EGCG (0.625–1 mg/ml) was also used to make a standard curve for determination of EGCG present in GTE. In our analysis, 20 µl of GTE (2.0 g%, *w/v*) was injected and fractionated on the column (C18 type, 150 mm × 4.6 mm, 5 µm particle size) capped with a guard column (C18 type, 10 mm × 4.7 mm, 5 µm particle size) (Agilent Technologies Inc., Santa Clara, CA, United States). The sample was then eluted isocratically with a mobile-phase solvent containing 0.05% H_2_SO_4_:acetonitrile:ethyl acetate (86:12:2, *v/v/v*) at a flow rate of 1.0 ml/min and the yield was measured at 280 nm. The EGCG concentration was determined with the use of a calibration curve produced from various concentrations of standard EGCG.

### 2.3 Animal care

Male heterozygous β−globin knockout (BKO, muβ^+/-^) mice (strain C57/BL6, aged 2–3 months, body weight (BW) 25–30 g) were kindly provided by the Thalassemia Research Center, Institute of Molecular Biosciences, Mahidol University (Salaya Campus), Nakorn Pathom Province, Thailand. The study protocol was approved of by the Animal Ethical Committee of the Faculty of Medicine, Chiang Mai University, Chiang Mai, Thailand (Protocol Number 19/2563). Mice were housed separately in polyethylene cages and acclimatized in a conventional clean room under standard conditions (23 ± 1°C and 40–70% humidity, 12 h day/12 h night cycle) for 14 days. They were fed with a normal diet (Mouse Feed Food Number CP082, Charoen Pokaphan Company, Bangkok, Thailand) containing carbohydrates (495.3 g), fat (83.7 g), protein (269.0 g), vitamins (65.4 g), and fiber (34.3 g). Mice were allowed to drink clean DW *ad libitum*.

### 2.4 Iron loading and treatment

In the absence of iron loading, wild type (WT) and BKO mice (*n* = 6 each) were intraperitoneally injected with 0.85% normal saline solution (NSS) for 20 days. The mice were allowed to equilibrate for further 23 days and were then treated with DI every day for 60 days. Similarly, BKO mice were induced to become iron overloaded by intraperitoneal (*ip*) injection of iron dextran (10 mg/d each) for 20 days according to our previously established method (Settapramote et al., 2021). Mice were then allowed to equilibrate for further 23 days. The iron dextran−loaded BKO mice were divided into four groups (*n* = 6 each) and treated with DI, GTE (50 mg EGCG equivalent/kg), DFP (50 mg/kg) and GTE (50 mg EGCG equivalent/kg) along with DFP (50 mg/kg) every day for 60 days. BW was recorded weekly for 4 months. At the end of the study, the mice were sacrificed using cervical dislocation and the heart blood was collected from the left ventricle and transferred into sodium-heparin anticoagulant coated tubes for measurement of RBC indices and plasma EPO, ERFE, and ferritin concentrations. Spleen, liver, and kidneys were collected, weighed and dissected for biochemical and pathological examination. The tissues were divided into two parts, of which one part was fixed in 10% (*v/v*) formaldehyde buffered in PBS for histochemical and histopathological investigations, and the other part was kept frozen at −80°C for analysis of tissue iron and lipid-peroxidation product content, as well as expression of *Epo* and *Erfe* genes as has been described below.

### 2.5 Hematological parameter assay

RBC indices were analyzed using an Auto Hematology Analyzer (Model Mindray BC 5300 Vet Analyzer, Medsinglong Company Limited, Guangdong, People's Republic of China) for animal blood testing at the Veterinary Diagnostic Laboratory, Small Animal Teaching Hospital, Faculty of Veterinary Medicine, Chiang Mai University.

### 2.6 Enzyme immunoassay of EPO, ERFE, and Ft concentrations

Plasma EPO concentrations were measured using the sandwich enzyme-linked immunosorbent assay (ELISA)technique (Noe et al., 1999) according to the manufacturer’s instructions. Briefly, diluting buffer, standard EPO and plasma (100 μl each) were added to each well that had been precoated with rabbit anti−mouse EPO. The specimens were then incubated at 37°C for 1 h. Then, biotin-conjugated anti−mouse EPO reagent (100 μl) was added to each well, and they were incubated at 37°C for 1 h and washed three times with washing buffer. Afterwards, horseradish peroxidase (HRP)−conjugated streptavidin reagent (100 μl) was added and the specimens were incubated at 37°C for 30 min. After being washed with washing buffer, tetramethylbenzidine (TMB)/hydrogen peroxide substrate solution (90 μl) was added and the specimens were incubated at 37°C for 10–20 min. Finally, 1 N sulfuric acid solution (50 μl) was added to every well in order to stop the reaction and the yellow−colored product was measured for optical density (OD) at a wavelength of 450 nm in a microplate reader, wherein the concentration of EPO in the plasma was determined from a calibration curve of standard EPO (6.25–400 pg/ml). A quality control assessment of the test indicated a sensitivity value of <2.68 pg/ml, as well as coefficients of the variable values for intra−assay precision and inter−assay precision at <10% and <12%, respectively. Similarly, plasma ERFE concentrations were measured using the sandwich ELISA technique (Kautz et al., 2015) according to the manufacturer’s instructions. In the assay, diluting buffer, standard ERFE and plasma were added to each well that had been precoated with rabbit anti−mouse ERFE and incubated for 1 h. Then, biotin−conjugated anti−mouse ERFE reagent was added and the specimens were incubated for 1 h. Afterwards, HRP−conjugated streptavidin reagent was added and the specimens were incubated for 30 min. TMB/hydrogen peroxide substrate solution was added and the specimens were incubated for 10–20 min. Finally, 1 N sulfuric acid solution was added to stop the reaction and the yellow−colored product was measured in terms of OD at 450 nm, wherein the concentration of ERFE in the plasma was determined from a calibration curve of standard ERFE (31.2–2,000 pg/ml). Likewise, plasma ferritin concentrations were measured using the sandwich ELISA technique (Conradie and Mbhele, 1980; Madersbacher and Berger, 2000) with slight modifications according to the manufacturer’s instructions. In the assay, diluting buffer, standard ferritin and plasma were added to each well that had been precoated with rabbit anti-mouse ferritin and incubated for 1 h. Then, biotin-conjugated anti−mouse ferritin reagent was added and the specimens were incubated for 1 h. Afterwards, HRP−conjugated streptavidin reagent was added and the specimens were incubated for 30 min. TMB/hydrogen peroxide substrate solution was added and the specimens were then incubated for 10–20 min. Finally, 1 N sulfuric acid solution was added to stop the reaction and the yellow−colored product was measured in terms of OD at 450 nm, wherein the concentration of ferritin in the plasma was determined from a calibration curve of standard ferritin (1.56–100 ng/ml). A quality control assessment of the test indicated a sensitivity value of <0.94 ng/ml, as well as coefficients of variation values for both intra-assay and inter-assay precision values at <10%.

### 2.7 Quantification of *Epo* mRNA expression

#### 2.7.1 RNA extraction

*Epo* mRNA were quantified in kidney tissues using the qRT−PCR method (Chomczynski and Mackey, 1995). Firstly, the kidney (100 mg wet weight) was dissected, homogenized with TRIzol reagent (1 ml) and incubated at room temperature for 5 min. Secondly, chloroform (200 µl) was added to the reaction mixture, and it was then incubated at room temperature for 5 min and centrifuged at 12,000 g, 4°C for 15 min. Thirdly, the aqueous−phase layer was transferred to a new tube, mixed with isopropanol (500 µl), then incubated at room temperature for 5 min and centrifuged at 12,000 g, 4°C for 10 min. Fourthly, RNA (white pellets) was collected, centrifuged-washed with 75% ethanol at 7,500g, 4°C for 5 min and dried. It was then resuspended in DEPC water (20 µL). RNA concentration and purity values were determined by NanoDrop spectrophotometer using wavelengths of 260 and 280 nm.

#### 2.7.2 cDNA synthesis

In assay, total RNA (1 µg) was reversely transcribed to complementary DNA (cDNA) using a Thermo Scientific RevertAid First Strand cDNA Synthesis kit according to the manufacturer’s protocol and instructions. Then, cDNA concentration and purity values were measured with a microvolume spectrophotometer (NanoDrop™, Thermo Fisher Scientific, Waltham, MA, United States) using the wavelengths of 260 and 280 nm.

#### 2.7.3 Real-time quantitative reverse transcription polymerase chain reaction (qRT−PCR)

Accordingly, qRT−PCR analysis of cDNA was performed using a Maxima SYBR Green/ROX qPCR kit on the Applied Biosystems™ QuanStudio™ 6 Flex real−time PCR instrument according to the manufacturer’s instructions. The primer sequences used in this study are presented in Table 1. The instrument was set up with a default thermal cycler protocol provided by the producer as follows: 95°C for 10 min, 95°C for 15 s, 58°C for 30 s and 72°C for 30 s for 40 cycles. For each PCR reaction, 1,000 ng of cDNA was used as a template. All analyses were carried out in triplicate. Relative quantities were presented in each sample, which were then assessed with the 2^−(ΔΔCt)^ method.

**TABLE 1:** List of primers used in qRT−PCR.

Primer name	Source	Forward primer sequence (5′–3′)	Reverse primer sequence (5′–3′)
*Epo*	Mouse	GCCAAGGAGGCAGAAAATGT	TACCCGAAGCAGTGAAGTGA
*Erfe*	Mouse	ATGGGGCTGGAGAACAGC	TGGCATTGTCCAAGAAGACA
*Hprt*	Mouse	CTGGTTAAGCAGTACAGCCCCAA	CAGGAGGTCCTTTTCACCAGC

Abbreviations: *Epo*, erythropoietin gene; *Erfe*, erythroferrone gene; qRT−PCR, quantitative reverse-transcription polymerase chain reaction; *Hprt*, hypoxanthine-phosphoribosyltransferase gene.

### 2.8 Quantification of splenic *Erfe* mRNA expression

Spleen *Erfe* mRNA extraction, cDNA synthesis and qPCR analysis were performed has been described in Section 2.7, with the use of the *Erfe* primer (Kautz et al., 2015).

### 2.9 Colorimetric determination of TIC

Iron contents in liver, spleen, and kidney tissues were stoichiometrically determined using the ferrozine colorimetric method (Koonyosying et al., 2018; Koonyosying et al., 2019). In principle, ferrozine-based assay can detect nonheme ferric and ferrous iron in the plasma, cells, and tissues following deproteinization with acidic solution, which has been determined to be very sensitive as indicated by atomic absorption spectroscopy in amounts ranging from between 0.2 and 30 nmol (Riemer et al., 2004). In the assay, the tissue samples (100 mg) were dried in a hot air oven at 70°C overnight and homogenized in a solution containing 50 mM phosphate buffer pH 2.8 (400 µl), BHT (50 ppm) (50 µl) and methanol in an ice bath using a hand homogenizer that had been previously washed with a hydrochloric acid:nitric acid (1:3 in molar ratio) solution. The homogenate was then vortex mixed with a protein precipitation solution (1 M HCl, 10% TCA in high-purity DI) at 95°C for 1 h, then cooled down to room temperature and centrifuged at 3,000 g, 4°C for 10 min. The supernatant (50 µl) was then mixed with the chromogenic solution (190 µl) containing 0.508 mM ferrozine, 1.5 M sodium acetate and 1.5% (*v/v*) TGA and incubated at room temperature for 30 min. Finally, the colored product solution was photometrically measured for optical density (OD) at 562 nm against the reagent blank. Iron concentrations of the tissues were determined from a calibration curve produced from different FAS concentrations (0–400 µM).

### 2.10 Pathological examination

#### 2.10.1 Hematoxylin and eosin (H & E) staining

Firstly, liver, spleen and kidney tissues were dissected, fixed in 10% (*v*/*v*) formalin solution, embedded in a paraffin box and cut using a microtome. Secondly, the tissue sections were deparaffinized and rehydrated sequentially in xylene, 100% (*v*/*v*) ethanol, 95% ethanol, 80% ethanol and DI. Thirdly, the sections were stained with hematoxylin solution (Harris hematoxylin combined with glacial acetic acid), rinsed with DI, destained with acidic ethanol (70% ethanol in diluted hydrochloric acid), counterstained with eosin solution (eosin phloxine) and then dehydrated with 95% ethanol, 100% ethanol and xylene sequentially. Slides were then covered using a per−mounting solution. The stained tissue sections were investigated under a digital light microscope (Nikon Instrument Inc., Minato, Tokyo, Japan) at a magnification of ×40 by an expert clinical pathologist, Dr. Sarawuth Kongkarnka, MD., at the Department of Pathology, Faculty of Medicine, Chiang Mai University.

#### 2.10.2 Perl’s staining

Perl’s Prussian blue (potassium ferrocyanide) reagent was used to stain the nonheme ferric iron content in the ferritin and hemosiderin, rather than in the ferrous iron in the labile iron pool that had been deposited in the tissues forming the Prussian blue pigment (a complex hydrated ferric ferrocyanide substance) (Parmley et al., 1978). This was visible microscopically (×400) and evaluated semi-quantitatively (from +1 to +4). Briefly, dissected liver, spleen, and kidney tissues were fixed with 10% (*v/v*) formalin, serially dehydrated once with 95% ethanol and twice with 100% ethanol, embedded in paraffin and stained with equal volumes of 20% (*v/v*) hydrochloric acid and 10% (*w/v*) potassium ferrocyanide for 20 min. Bright blue pigments were examined under a light microscope and the blue granules were recorded using a digital camera (Koonyosying et al., 2018). Iron distribution scores of Perl’s ferrocyanide-stained liver, spleen, and kidney tissues were evaluated using the method previously described by Yatmark and coworkers (Yatmark et al., 2014). With the use of a light microscope employing ×40 magnification, the visualized image of the stained tissue was snapped at 200 µm long using ImageScope Version 12.3.2.5030 Software, while every ten non-overlapping fields were analyzed using ImageJ Ver. 1.53k Software. For the purposes of interpretation, the original digital images were converted into eight-bit black−and−white images and their intensity was present between 0 and 255. The single pixels were then analyzed in order to produce the appropriate grayscale values and a histogram.

### 2.11 Measurement of lipid−peroxidation products

Lipid-peroxidation products, such as malondialdehyde, were quantified using the colorimetric TBARS method that had been previously described (Koonyosying et al., 2018). The tissue homogenates were deproteinized with 10% TCA solution containing 50 mg/L BHT (220 µL) and centrifuged at 3,000 g for 10 min. After centrifugation, the supernatant (50 µl) was incubated with a chromogenic solution containing 0.44 M H_3_PO_4_ (150 µl) and 0.6% (*w/v*) TBA (100 µl) at 90°C for 30 min, then cooled down at room temperature and OD was photometrically measured at 540 nm. TBARS concentrations were determined from a calibration curve produced from different TMP concentrations (0–100 µM).

### 2.12 Statistical analysis

Results were analyzed using the IBM SPSS Statistics 22 Program (version 18) and expressed as mean ± SEM values. Statistical significance was determined using one-way analysis of variance (ANOVA) with *post hoc* Bonferroni, for which *p* < .05 was considered significantly different.

## 3 Results

### 3.1 Green tea extract

**FIGURE 1:**
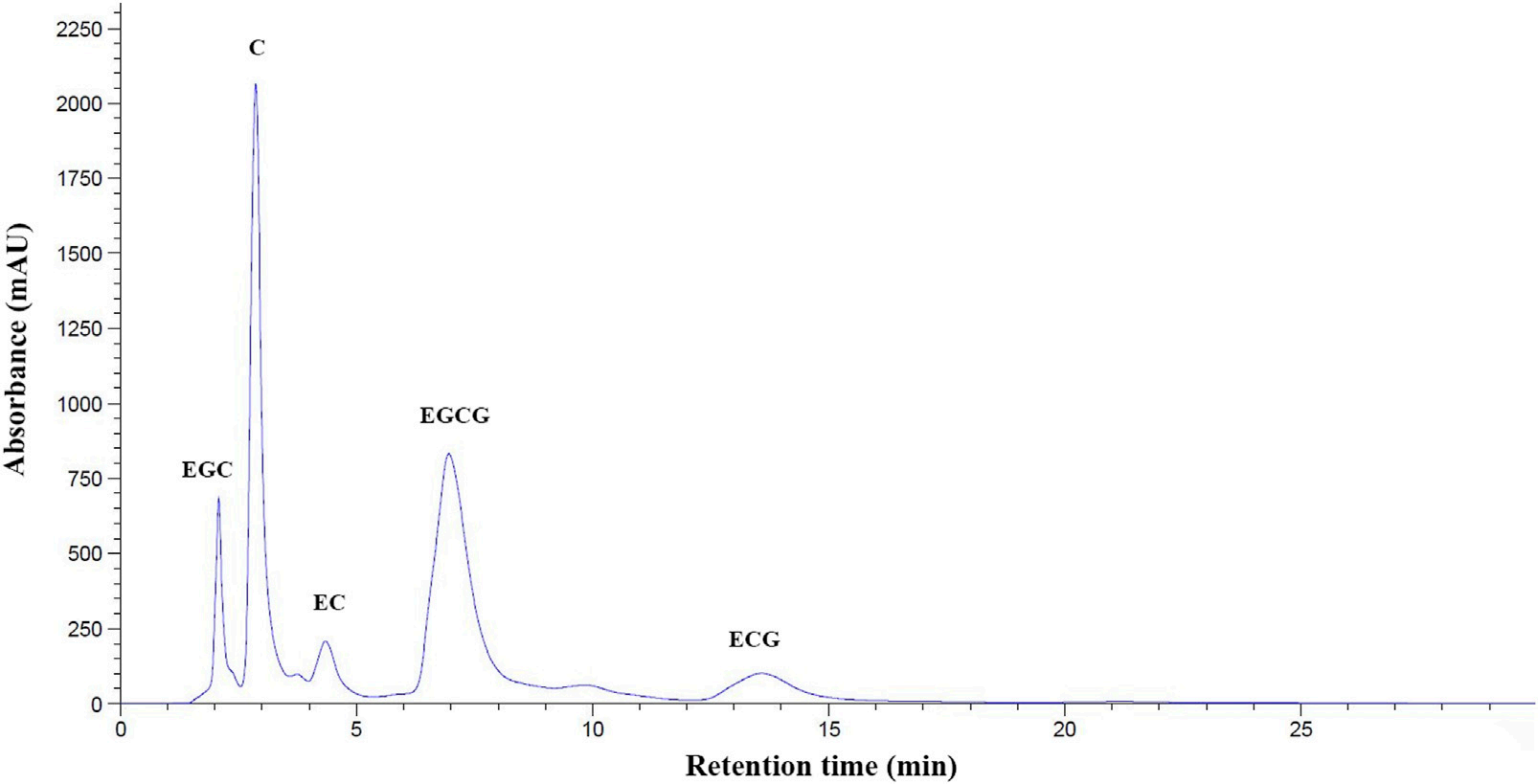
HPLC−DAD profile of polyphenolic catechins in GTE. Abbreviations: C = catechin, ECG = epicatechin-3-gallate, EGC = epigallocatechin, EGCG = epigallocatechin 3-gallate, GTE = green tea extract, HPLC−DAD = high performance liquid chromatography coupled with diode−array detection, mAU = milli-absorbance unit, OD = optical density.

With the use of high-performance liquid chromatography coupled with the diode-array detection (HPLC−DAD) technique, Standard EGC, C, EC, EGCG, and ECG were sequentially eluted at the retention time (RT) of 2.22, 3.05, 4.31, 7.35, and 13.75 min. Consistently, our hot−water GTE was found to contain at least five polyphenolic catechins, of which EGCG was eluted at a retention time (TR) of 7.39 min. This corresponded to that of the authentic EGCG (10 mg/ml) (Figure 1). According to calculations of the standard curve of EGCG (0–1 mg/ml), we orally administered GTE comprised of 55.48 ± 0.07 mg EGCG/g at 50 mg EGCG equivalent/kg body weight (BW) for either monotherapy or combined therapy with DFP in BKO thalassemic mice throughout the course of this study.

### 3.2 Red blood cell indices

BKO thalassemia mice were found to be mildly anemic due to ineffective erythropoiesis. When initiating this study, there was one group of wild-type C57/BL6 (WT) mice (*n* = 6) and five groups of β−gene knockout C57/BL6 (BKO) mice (*n* = 6 each) that were prepared with and without iron dextran loading along with the interventions. Having finished the intraperitoneal (*ip*) injections of iron dextran, the alive mice in those groups were treated with deionized water (DI), GTE, DFP and a combination of GTE and DFP. At the end of the study, the mice (*n* = 5, 6, 6, and 6, respectively) in those groups were sacrificed and their blood samples and tissues were analyzed. These mice mimicked thalassemia intermedia patients by exhibiting lower hemoglobin (Hb) levels, lower packed RBC (Hct) levels, and lower RBC numbers than WT mice (*p* < .05). These levels were also lower than those within the reference resource but were non−significantly higher in terms of the mean corpuscular hemoglobin (MCH), mean corpuscular hemoglobin concentration (MCHC) and the RBC distribution width (RDW) values (Table 2). As a consequence of *ip* injections of iron dextran into BKO mice at a dose of 10 mg each, there were increases in RBC numbers, as well as the Hct, and RDW (*p* < .05) levels; while decreasing MCV, MCH (*p* < .05) and MCHC (*p* < .05) levels were observed. Accordingly, a nonsignificant change in Hb level was observed when compared with the BKO mice without iron loading. However, treatments on iron dextran-loaded BKO mice with GTE (50 mg EGCG equivalent/kg), DFP (50 mg/kg), and a combination of the two compounds did not significantly change all RBC index levels when compared with the DI group of the iron dextran−loaded BKO mice.

**TABLE 2:** RBC indices of WT mice and iron dextran–injected BKO mice that were orally administered with DI, GTE (50 mg EGCG equivalent/kg), DFP (50 mg/kg) and GTE (50 mg EGCG equivalent/kg) in conjunction with DFP (50 mg/kg) for a period of two months. Data are expressed as mean ± standard error of the mean (SEM) values. Accordingly, ^&^ *p* < 0.05 when compared with WT mice; ^♣^ *p* < 0.05, ^♣♣^ *p* < 0.01 when compared with DI in iron dextran–loaded BKO mice.

Group	Iron loading	Treatment	RBC number (x 10^6^/µL)	Hb (g/dL)	Hct (%)	MCV (fL)	MCH (pg)	MCHC (g/dL)	RDW (%)
Reference			6.5 - 11.5	11.0 - 15.5	35.0 - 50.0	41.0 - 52.0	(13.0 - 18.0)	(30.0 - 35.0)	(11.0 - 17.0)
WT (n = 5)	NSS	DI	7.64±0.67	12.22±0.89	38.83±3.78	50.55±0.98	16.13±0.34	32.03±1.30	15.62±0.47
BKO (n = 5)	NSS	DI	4.59±0.05^&^	8.40±0.10^&^	21.70±0.18^&^	47.3±0.70	18.1±0.26	38.3±0.27	18.1±0.18
BKO (n = 5)	iron dextran	DI	7.61±0.05	8.20±0.80	30.80±2.35	40.45±0.25	10.80±0.10^♣^	26.65±1.05^♣^	35.40±0.30^♣♣^
BKO (n = 3)	iron dextran	GTE	5.83±0.65	7.33±0.79	26.07±2.36	45.10±1.25	12.80±1.65	28.07±1.52	30.90±3.86
BKO (n = 4)	iron dextran	DFP	7.53±0.21	8.00±0.12	30.10±2.00	40.00±0.70	10.60±1.20	26.60±1.50	34.40±1.40
BKO (n = 3)	iron dextran	GTE + DFP	7.54±0.19	8.20±0.30	31.70±1.10	42.10±0.10	10.90±1.30	25.80±1.10	36.50±1.60

Abbreviations: BKO, beta–globin knockout; DFP, deferiprone; DI, deionized water; GTE, green tea extract; Hb, hemoglobin; Hct, hematocrit; MCH, mean corpuscular hemoglobin; MCHC, mean corpuscular hemoglobin concentration; MCV, mean corpuscular volume; NSS, normal saline solution; RBC, red blood cell; RDW, red cell distribution width; WT, wild type.

### 3.3 Body weight, tissue weight and tissue weight index

**FIGURE 2:**
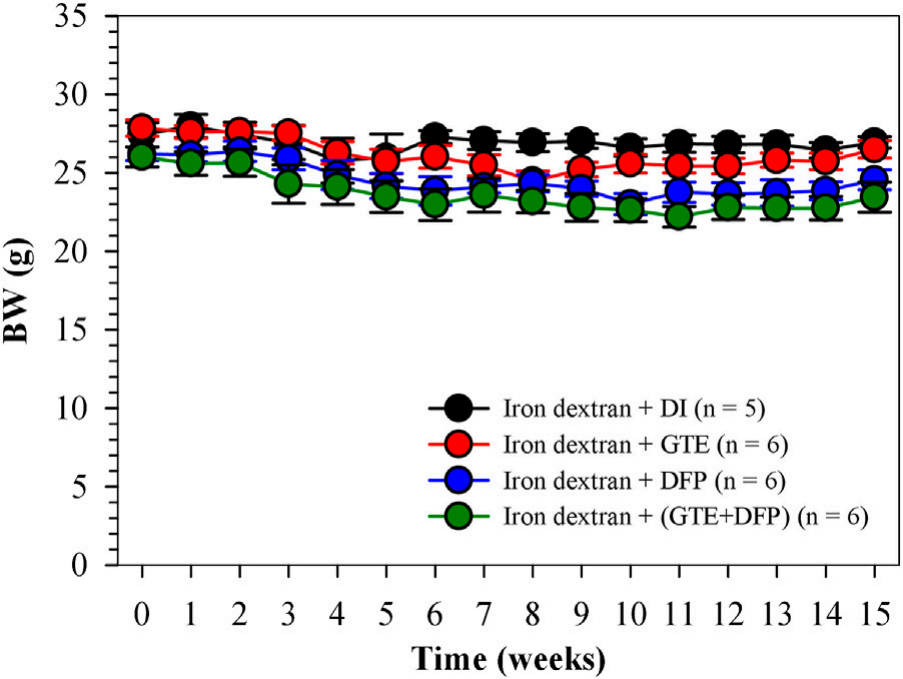
BW of iron dextran loaded−BKO mice that had been treated with DI, GTE (50 mg EGCG equivalent/kg), DFP (50 mg/kg) and GTE (50 mg EGCG equivalent/kg) in conjunction with DFP (50 mg/kg) for 2 months. Data are expressed as mean ± SEM values. Abbreviations: BKO = β-globin gene knockout, BW = body weight, DFP = deferiprone, DI = deionized water, EGCG = epigallocatechin 3-gallate, GTE = green tea extract, SEM = standard error of the mean.

Over a 1−month period of iron−dextran loading, followed by a 3−month intervention period (2 months for treatment and 1 month for equilibration), their body weight was recorded weekly. As is shown in Figure 2, BW values of all of the mice groups were slightly decreased when compared with the pre-treatment levels, while the changes were not found to be significantly different among mice of the treatment groups when compared with the mice that underwent DI treatments.

**FIGURE 3:**
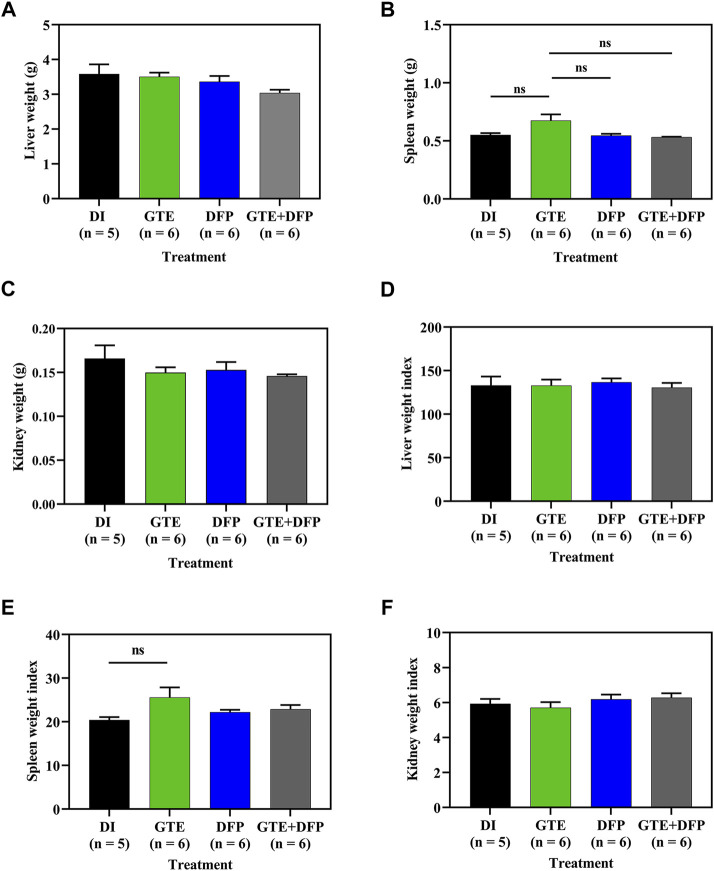
Wet weight and weight index values of the liver **(A,D)**, spleen **(B,E)**, and kidneys **(C,F)** of iron dextran–loaded BKO mice treated with DI, GTE (50 mg EGCG equivalent/kg), DFP (50 mg/kg) and GTE (50 mg EGCG equivalent/kg) in conjunction with DFP (50 mg/kg) for a period of 2 months. Data are expressed as mean ± SEM values. Abbreviations: BKO = β–globin gene knockout, DFP = deferiprone, DI = deionized water, EGCG = epigallocatechin 3–gallate, GTE = green tea extract, NS = nonsignificant, SEM = standard error of the mean.

As is shown in Figures 3A−C, the wet weights of the liver, spleen, and kidneys obtained from iron dextran−induced BKO mice that received GTE and DFP monotherapy, and a combination of the two, for 60 days did not change significantly, whereas the combination treatment did tend to result in a decrease in liver weights in the mice. With regard to the organ weight index (WI), which is presented as a ratio of tissue wet weight and BW, in conjunction with monotherapy involving GTE or DFP, the combination therapy did not significantly influence WI values of the liver, spleen, and kidneys of iron-loaded BKO mice when compared with those that had received the DI treatment. Remarkably, no significant differences were observed among the mice receiving all treatments (Figures 3D-F).

### 3.4 Levels of plasma EPO, ERFE, and Ft

**FIGURE 4:**
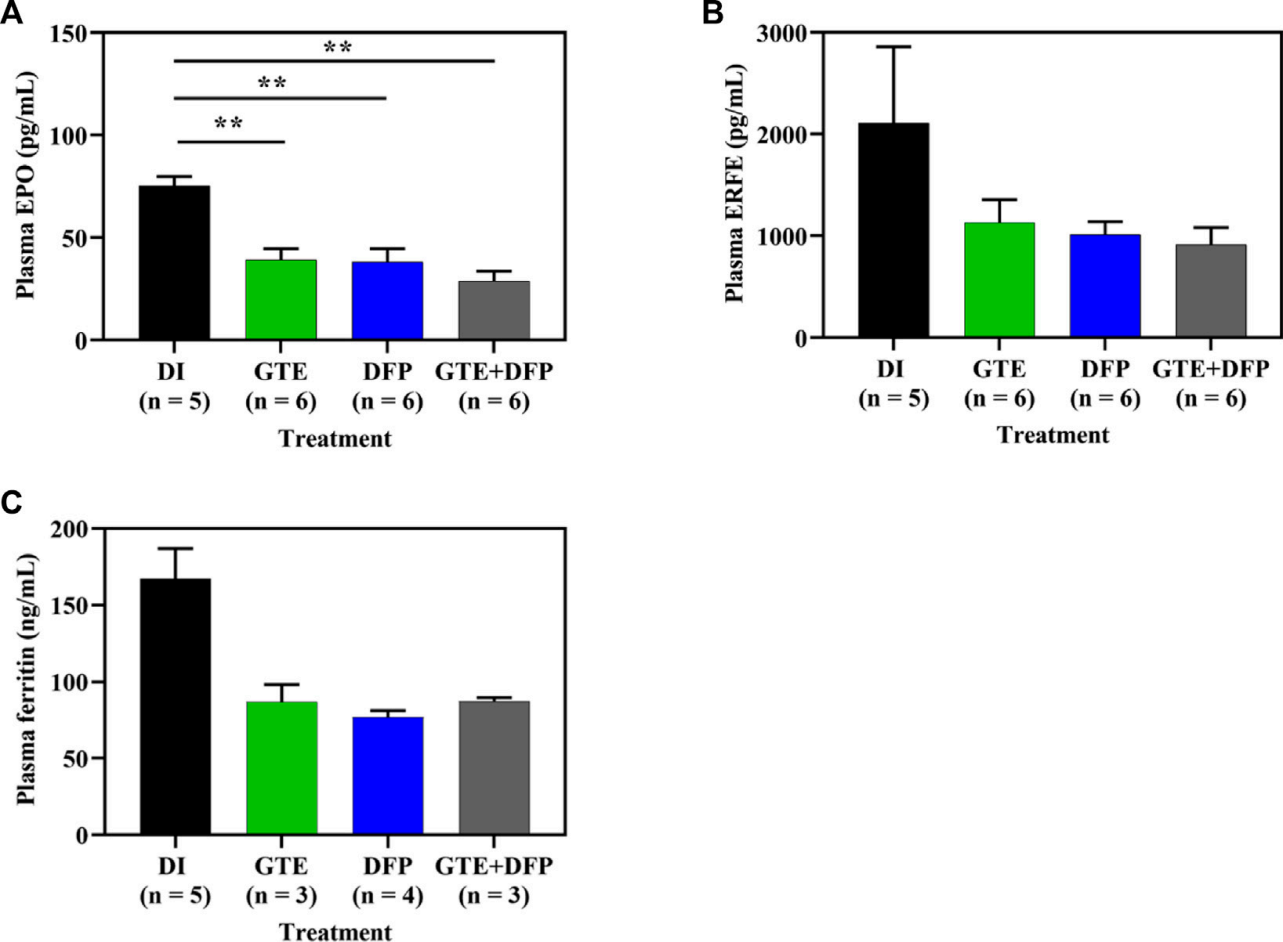
Plasma EPO **(A)**, ERFE **(B)** and Ft **(C)** concentrations in iron dextran-loaded BKO mice treated with DI, GTE (50 mg EGCG equivalent/kg), DFP (50 mg/kg) and GTE (50 mg EGCG equivalent/kg) in conjunction with DFP (50 mg/kg) for a period of 2 months. Data are expressed as mean ± SEM values. Accordingly, ^**^ *p* < 0.01 when compared with DI. Abbreviations: BKO = β-globin gene knockout, DFP = deferiprone, DI = deionized water, EGCG = epigallocatechin 3-gallate, EPO = erythropoietin, ERFE = erythroferrone, GTE = green tea extract, SEM = standard error of the mean.

Surprisingly, treatments involving GTE, DFP, and a combination of the two resulted in decreased levels of plasma EPO (*p* < 0.01) (Figure 4A), ERFE (Figure 4B) and Ft (Figure 4C) in iron−loaded BKO mice when compared with mice in the control DI group. No significant differences were observed among mice in the treatment groups.

### 3.5 *Epo* and *Erfe* mRNA expression

**FIGURE 5:**
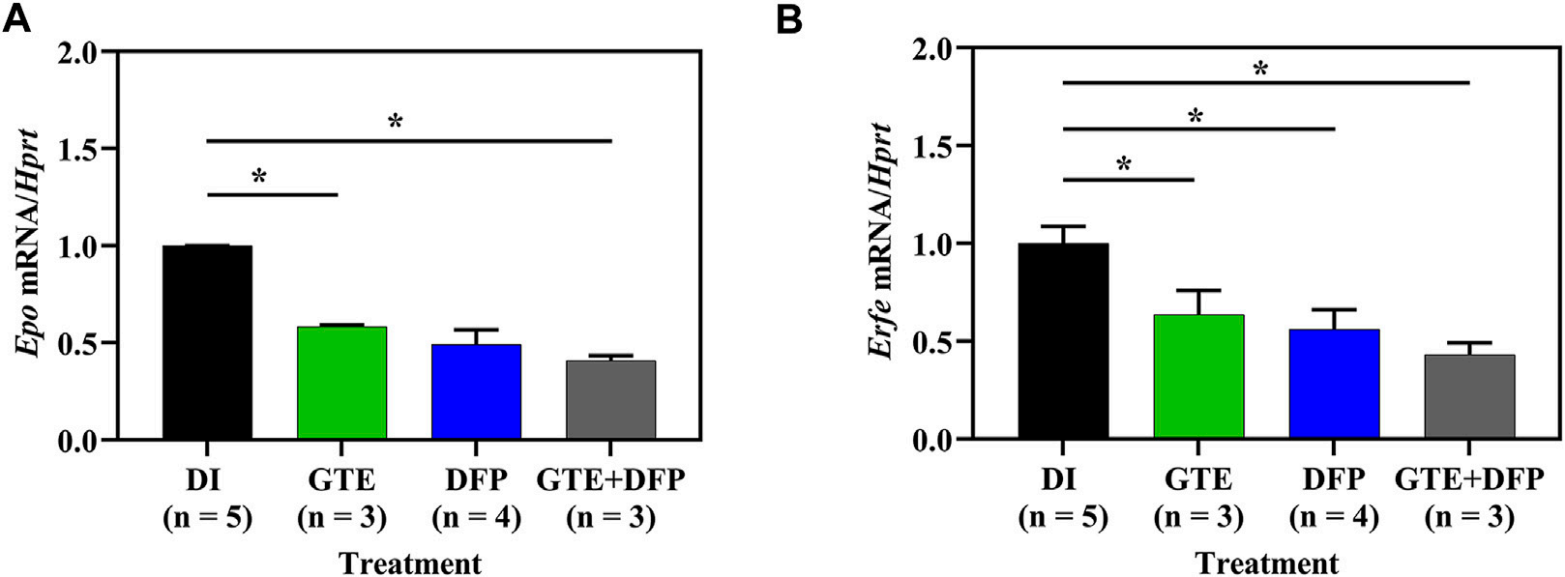
Herein, *mRNA* levels of *Epo* **(A)** and *Erfe* **(B)** taken from kidney and spleen tissues, respectively that had been collected from iron dextran−loaded BKO mice treated with DI, GTE (50 mg EGCG equivalent/kg), DFP (50 mg/kg) and GTE (50 mg EGCG equivalent/kg) in conjunction with DFP (50 mg/kg) for a period of 2 months. Data are expressed as mean ± SEM values. Accordingly, ^*^ *p* < .05 when compared with DI. Abbreviations: BKO = β-globin gene knockout, DFP = deferiprone, DI = deionized water, EGCG = epigallocatechin 3-gallate, *Epo* = erythropoietin gene, *Erfe* = erythroferrone gene, GTE = green tea extract, SEM = standard error of the mean.

Apparently, *Epo* mRNA levels in iron−loaded BKO mice decreased after treatments of GTE (*p* < .05), DFP and a combination (*p* < .05) of the two when compared with mice of the DI treatment group, while no significant differences were observed among all mice receiving compound treatments (Figure 5A). In a similar fashion, E*rfe* mRNA levels in iron−loaded BKO mice were significantly decreased by treatments of GTE, DFP, and a combination of the two when compared with mice that had received the DI treatment (Figure 5B). In both cases, the combined treatment seemed to be slightly more effective than the monotherapy but non-significantly.

### 3.6 Tissue iron content

**FIGURE 6:**
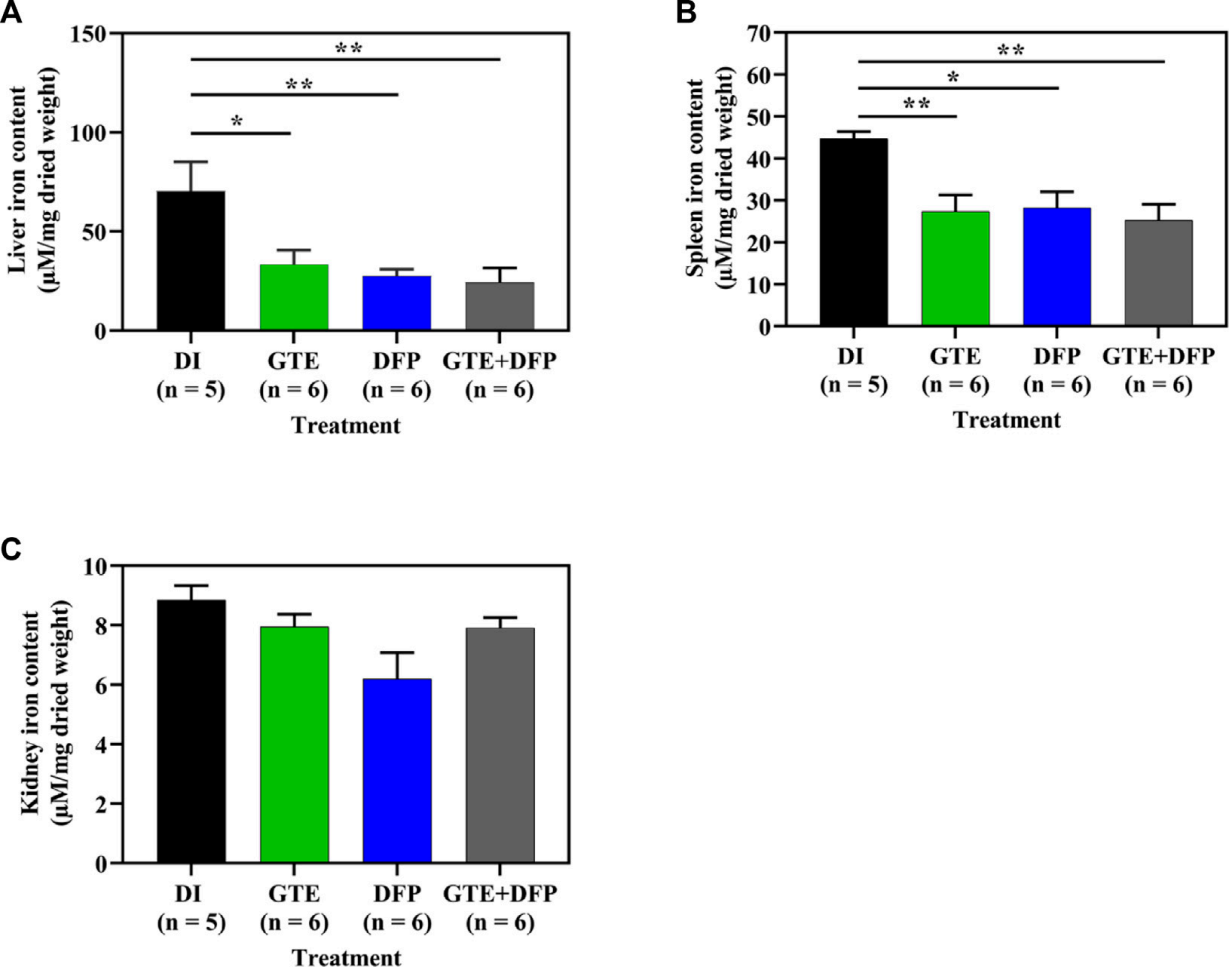
Iron content in the liver **(A)**, spleen **(B)**, and kidney **(C)** tissue obtained from iron dextran–loaded BKO mice treated with DI, GTE (50 mg EGCG equivalent/kg), DFP (50 mg/kg) and GTE (50 mg EGCG equivalent/kg) in conjunction with DFP (50 mg/kg) for a period of 2 months. Data are expressed as mean ± SEM values. Accordingly, ^*^ *p* < .05, ^**^ *p* < .01 when compared with DI. Abbreviations: BKO = β–globin gene knockout, DFP = deferiprone, DI = deionized water, EGCG = epigallocatechin 3–gallate, GTE = green tea extract, SEM = standard error of the mean.

Importantly, GTE and DFP monotherapy, and the combination therapy significantly decreased the amount of iron accumulating in the livers and spleens of iron-loaded BKO mice when compared with BKO mice that had received DI treatment. However, there was no additive effect in the mice receiving a combination therapy of GTE and DFP when compared with the DI and the monotherapy groups. No significant differences were observed among mice of the treatment groups either (Figures 6A−C).

### 3.7 Tissue iron distribution

**FIGURE 7:**
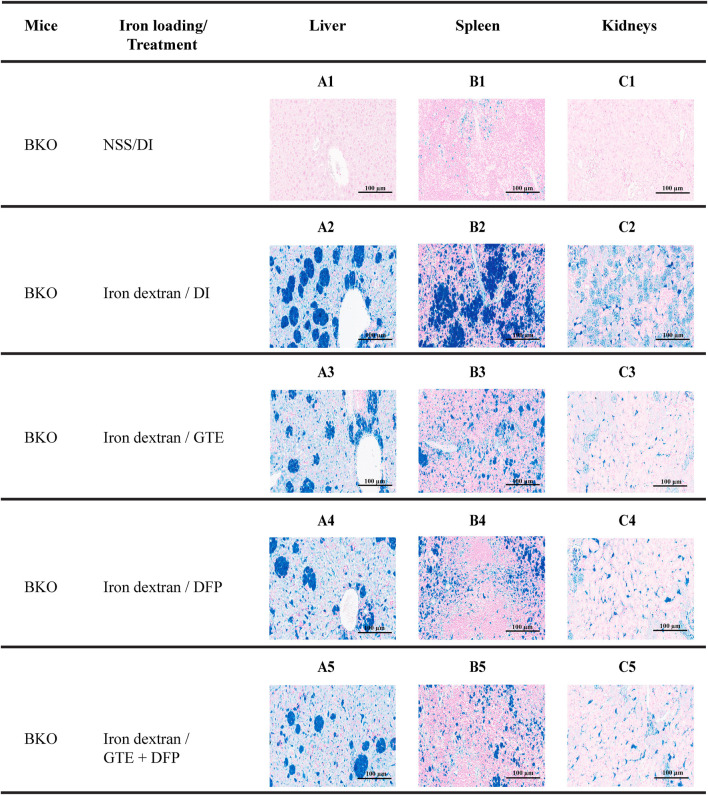
Perl’s stained iron distribution levels in the liver (A1-A5), spleen (B1-B5), and kidneys (C1-C5) of BKO mice with or without iron dextran loading. These mice had been treated with DI, GTE (50 mg EGCG equivalent/kg), DFP (50 mg/kg) and GTE (50 mg EGCG equivalent/kg) in conjunction with DFP (50 mg/kg) for a period of 2 months. Abbreviations: BKO = β–globin gene knockout, DFP = deferiprone, DI = deionized water, EGCG = epigallocatechin 3–gallate, GTE = green tea extract.

**FIGURE 8:**
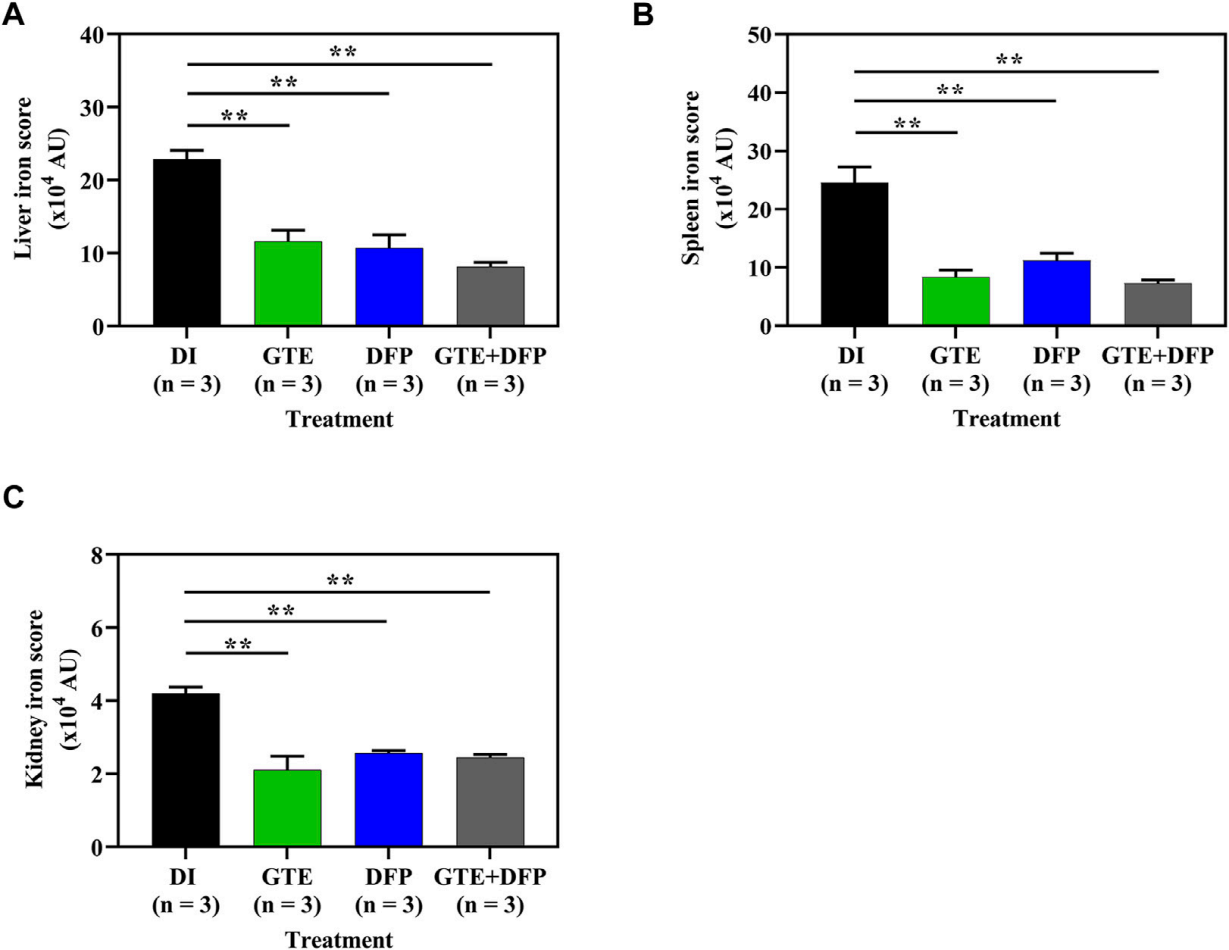
Iron distribution scores in the liver **(A)**, spleen **(B)**, and kidney **(C)** tissues obtained from iron dextran−loaded BKO mice treated with DI, GTE (50 mg EGCG equivalent/kg), DFP (50 mg/kg) and GTE (50 mg EGCG equivalent/kg) in conjunction with DFP (50 mg/kg) for a period of 2 months. Data are expressed as mean ± SEM values. Accordingly, ^**^ *p* < 0.01 when compared with DI. Abbreviations: AU = arbitrary unit, BKO = β−globin gene knockout, DFP = deferiprone, DI = deionized water, EGCG = epigallocatechin 3−gallate, GTE = green tea extract, SEM = standard error of the mean.

According to semi−quantitative analysis, histochemical Perl’s stained iron distributions that appeared as Prussian blue granules in the liver, spleen, and kidney tissues obtained from iron-loaded BKO mice are shown in Figure 7. The results implied that *ip* injections of iron dextran increased the iron depositions in these tissues. This occurred to the highest degree in the liver, followed by the spleen and the kidneys, respectively. During the intervention period, tissue iron contents were decreased markedly by treatments of GTE, DFP, and the combined GTE and DFP treatment when compared with mice of the DI treatment group. Regarding the scanning of the blue granules, iron scores of the liver, spleen, and kidneys were apparently increased in the iron-loaded groups when compared to mice that had not undergone iron loading. GTE and DFP monotherapy and the combination treatment markedly decreased iron deposition values in the liver, spleen, and kidneys when compared with mice in the DI group; however, no differences were observed among all mice of the treatment groups (Figures 8A−C).

### 3.8 Tissue and plasma TBARS concentrations

**FIGURE 9:**
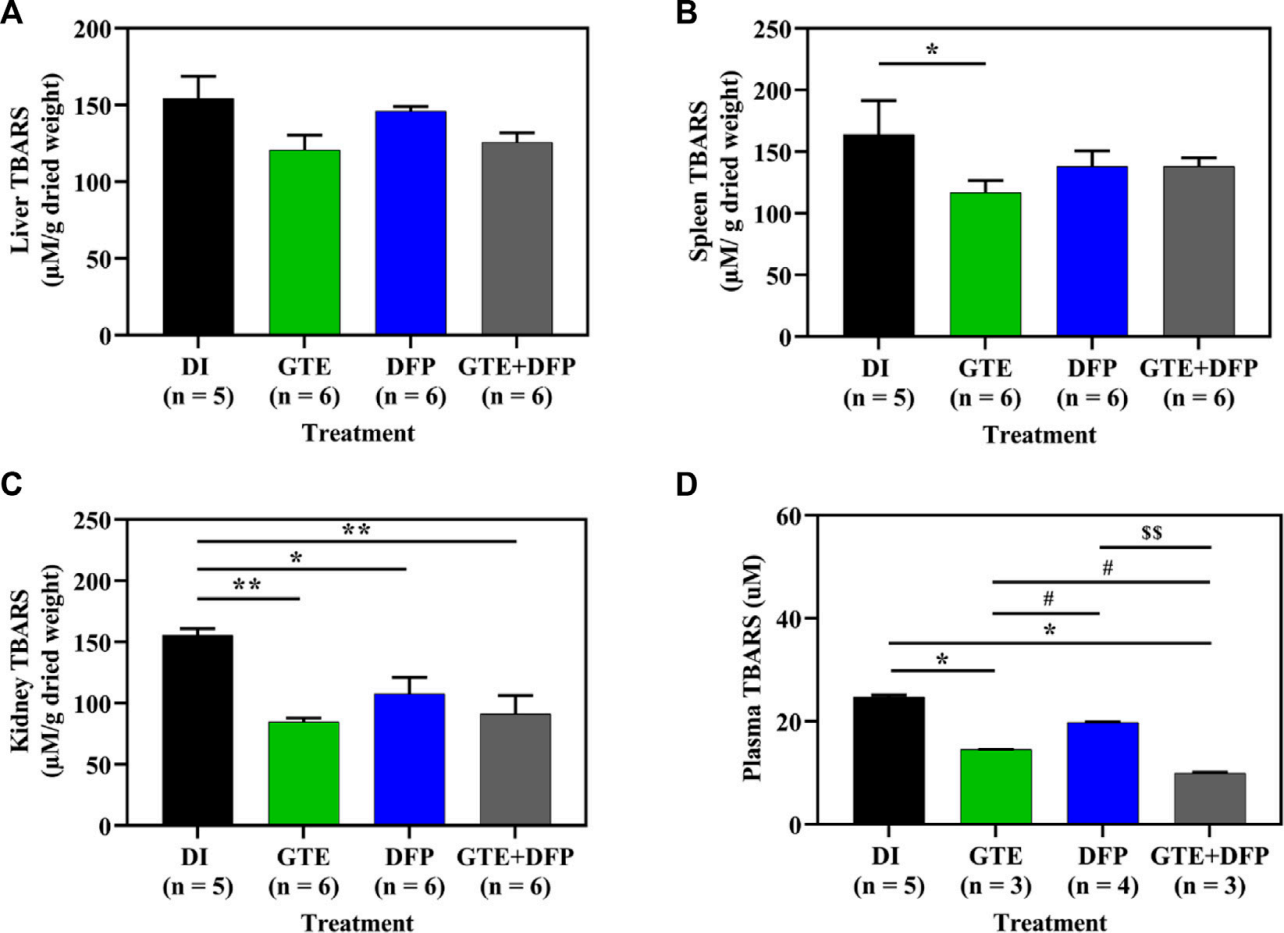
TBARS contents in the liver **(A)**, spleen **(B)**, kidney **(C)** tissues and pooled plasma **(D)** collected from iron dextran–loaded BKO mice that had been treated with DI, GTE (50 mg EGCG equivalent/kg), DFP (50 mg/kg) and GTE (50 mg EGCG equivalent/kg) in conjunction with DFP (50 mg/kg) for a period of 2 months. Data are expressed as mean ± SEM values. Accordingly, ^*^ *p* < .05, ^**^ *p* < .01 when compared with DI; ^#^ *p* < .05 when compared between GTE and DFP or GTE + DFP; ^$$^ *p* < .01 when compared between DFP and GTE + DFP. Abbreviations: BKO = β–globin gene knockout, DFP = deferiprone, DI = deionized water, EGCG = epigallocatechin 3–gallate, GTE = green tea extract, SEM = standard error of the means, TBARS = thiobarbituric acid–reactive substances.

Consistently, lipid−peroxidation products, such as TBARS, were presented in the liver, spleen, and kidney tissue of iron−loaded BKO mice, while TBARS levels decreased in mice that received treatments of GTE and DFP, and in those receiving a combination of the two compounds (Figures 9A−D). Moreover, plasma TBARS levels were significantly decreased after treatment with GTE, DFP and a combination of the two, whereas no significant differences were observed among all mice of the treatment groups.

### 3.9 Histopathological examination

**FIGURE 10:**
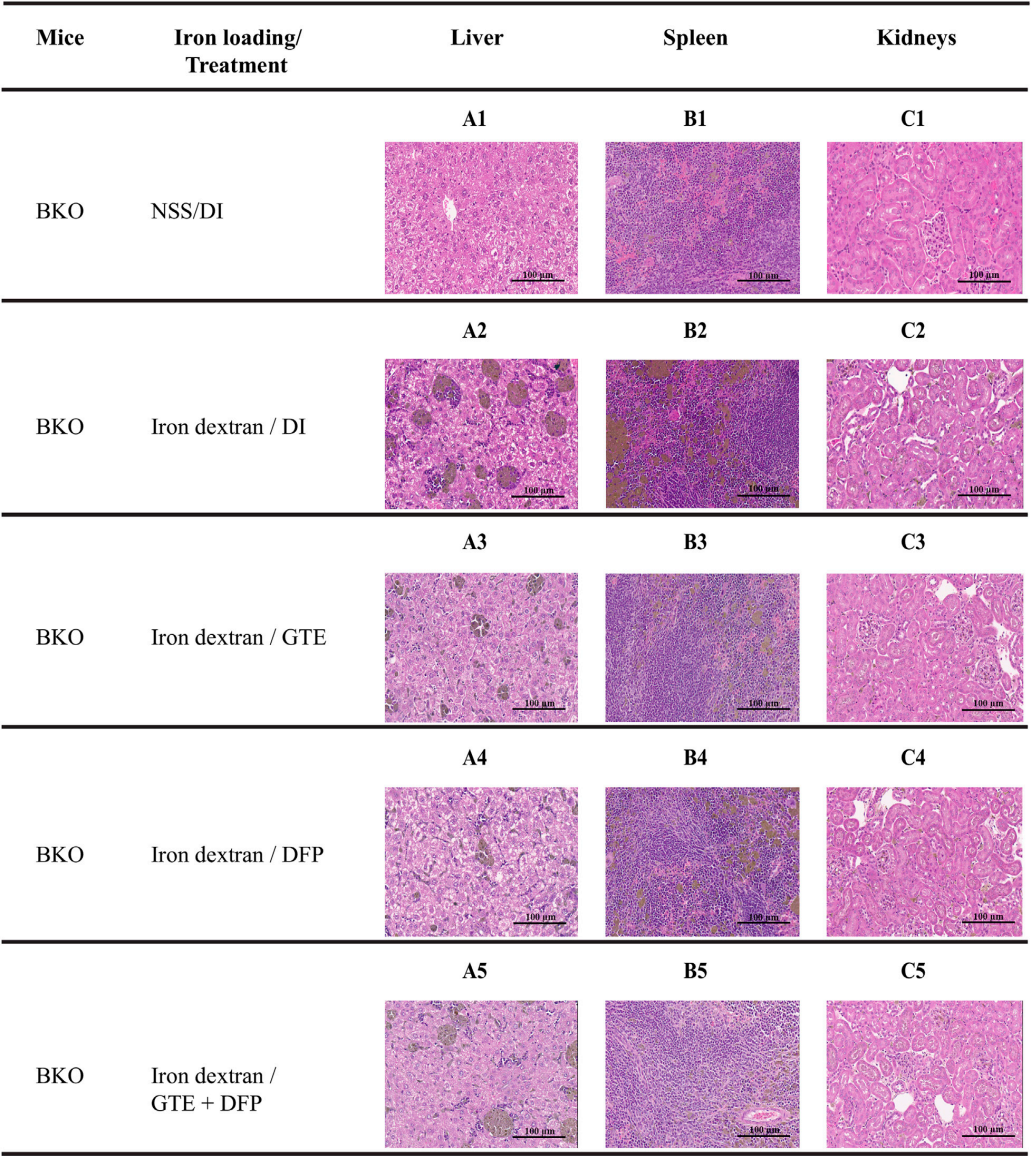
Hematoxylin and eosin staining of the liver **(A1-A5)**, spleen **(B1-B5)** and kidney **(C1-C5)** tissues from BKO mice with or without iron dextran loading. These mice had been treated with DI, GTE (50 mg EGCG equivalent/kg), DFP (50 mg/kg) and GTE (50 mg EGCG equivalent/kg) in conjunction with DFP (50 mg/kg) for a period of 2 months. Abbreviations: BKO = β–globin gene knockout, DFP = deferiprone, DI = deionized water, EGCG = epigallocatechin 3–gallate, GTE = green tea extract.

Unremarkable hepatic parenchymal tissue in a control DI group (Figure 10A1) showed intact hepatocytes with eosinophilic granular cytoplasm and basophilic uniform nuclei. Apparently, multiple aggregates of iron-laden macrophages in the sinusoids and scattered iron-laden hepatocytes were detected in the liver from iron dextran−loaded BKO mice, indicating stored iron in the form of ferritin, heme and lysosomal hemosiderin (Figure 10A2). A similar pathologic change was observed in the spleen (Figure 10B2) compared to non-iron overloaded group (Figure 10B1). When compared with mice of the DI treatment group, the iron deposition was decreased in the GTE, DFP and GTE + DFP−treated groups (Figures 10A3–A5, respectively), evidenced by fewer macrophage aggregation and decreased intensity of iron pigment. A similar effect was also demonstrated in the spleen (Figure 10B3–B5). For kidneys (Figures 10C1−C5), an iron deposit was observed in renal tubular cells with minimal to a mild degree.

## 4 Discussion

Green tea is a popular beverage around the world and can promote a range of beneficial functions in the human body. Tea (*C. sinensis*) leaves contain high amounts of polyphenols and aluminum. Green tea is produced from fermented tea leaves using a conventional green tea-processing machine. Tea can also be produced by heating leaves in a microwave oven, streaming, roasting and by ultrasonication to inactivate the inherent polyphenol oxidase (Srichairatanakool et al., 2006; Cajka et al., 2012; Luo et al., 2020; Thi Anh Dao et al., 2021). Among these processes, we found that microwave heating is the cheapest method and most capable of producing GTE rich in polyphenolic catechins including catechin, gallocatechin−3−gallate, epicatechin, epigallocatechin, epicatechin gallate (ECG) and EGCG, of which EGCG is known to be the most abundant (Srichairatanakool et al., 2006; Thephinlap et al., 2007). In terms of health benefits, GTE exerts various important antioxidant, free-radical−scavenging, anti−thromboxane, anti−cancer, cancer chemo−preventive and metal−chelating activities (Ali et al., 1990; Ho et al., 1992; Srichairatanakool et al., 2006; Santos et al., 2021). We had previously determined that GTE products decreased redox iron levels, such as those of non−transferrin bound iron (NTBI) and labile plasma iron, in the plasma levels of thalassemia patients. Furthermore, excessive amounts of iron and ROS in the iron−overloaded cells of β−thalassemic mice and β−thalassemia patients could possibly be decreased through the ROS−scavenging and iron−chelating actions of EGCG and ECG (Thephinlap et al., 2007; Ounjaijean et al., 2008; Saewong et al., 2010; Koonyosying et al., 2018; Koonyosying et al., 2019; Koonyosying et al., 2020b). Ineffective erythropoiesis, chronic anemia and iron overload are the major characterizations of both non−transfusion and transfusion−dependent β−thalassemia patients. These have been classified as non−transfusion−dependent thalassemia (NTDT) and transfusion−dependent thalassemia (TDT), respectively (Mishra and Tiwari, 2013). Accordingly, iron chelators are administered to thalassemia patients to reduce the deposition of excessive iron in their bodies, even though there are some known side effects (Viprakasit and Origa, 2014). Antioxidants, such as vitamin C, vitamin E and N−acetyl cysteine, are usually given to these patients along with iron chelators to relieve the state of oxidative stress. Herein, GTE was prepared using our established method, which is known to contain catechins, particularly EGCG, and was used throughout the course of this study (Srichairatanakool et al., 2006; Koonyosying et al., 2018).

In controversy, green tea polyphenols can interfere with the duodenal absorption of dietary iron which will result in iron−deficiency anemia in healthy people; inversely, the compounds exert an iron−chelating activity that can improve iron overload-associated oxidative stress in thalassemia patients. To induce iron overload in mice, *ip* injections of iron dextran are more controllable and require shorter periods of time than it takes to administer a ferrocene-supplemented diet (Saewong et al., 2010; Koonyosying et al., 2018; Settapramote et al., 2021). In this study, the nutraceutical effects of GTE were investigated in an attempt to address ineffective erythropoiesis, anemia and iron overload−related complications in β−thalassemia patients. A previous study has reported that the consumption of GTE increased serum aluminum levels but decreased blood Hb levels and tissue iron contents significantly in a tea dose−dependent manner in rats. This was possibly due to the competitive absorption of iron with aluminum by the duodenum (Marouani et al., 2007). Surprisingly, the oral administration of an acai (*Euterpe oleracea* Mart) extract containing polyphenols, iron, and vitamin E for 4 days significantly increased RBC numbers, as well as Hb, Hct and serum EPO levels. This administration also upregulated *Epo* gene levels in the kidneys of mice, which implied enhanced erythropoiesis (Shibuya et al., 2020). Interestingly, treatments of 10 nM EGCG were found to improve the survival rates of human CD34^+^ hematopoietic stem/progenitor cells that had been exposed to X−ray irradiation, which was suggestive of the protective effects of erythropoiesis (Monzen and Kashiwakura, 2012). Due to ineffective erythropoiesis and chronic anemia, β−thalassemia patients have experienced bone marrow expansion and extramedullary erythropoiesis in the spleen, liver, lungs, kidneys, and peritoneal cavity (Kim, 2010; Nienhuis and Nathan, 2012). In the present study, we have found that the gavage administration of GTE (50 mg EGCG equivalent/kg) to iron-loaded BKO mice for 2 months increased the number of RBCs, as well as the Hct and RDW values, while also decreasing MCV, MCH and MCHC values. Nonetheless, the GTE treatment did not produce any significant changes in the RBC, Hct, MCV, MCH, and MCHC values in iron dextran−loaded BKO mice when compared with the BKO mice that had not undergone iron loading. Thus, this action did not influence the Hb levels of iron dextran−loaded BKO mice suggesting that GTE could enhance erythropoietic activity in BKO mice with mild anemia. In addition, body weight and weight indices of the liver, spleen, and kidneys of BKO mice were not significantly different in the groups that had been treated with GTE, DFP, and a combination of the two when compared with mice in the DI group and mice of all treatment groups.

EPO production is activated under conditions of hypoxia and anemia to accelerate RBC production (Amer et al., 2010). In addition, treatments with EPO and certain drugs, such as hydroxyurea, hydroxybutyrate and DFP, will increase the production of fetal Hb and lead to the subsequent switching to adult Hb in thalassemia patients. In fact, serum EPO levels in β−thalassemia patients with iron overload are higher than those in healthy people (Nisli et al., 1997; Chaisiripoomkere et al., 1999). Likewise, the administration of recombinant EPO was found to decrease iron overload in many tissues; particularly in the livers of patients with hereditary hemochromatosis (De Gobbi et al., 2000). Moreover, Pootrakul and colleagues have reported that the treatment of DFP (25–50 mg/kg/d) significantly reduced levels of serum Ft, NTBI, TBARS and liver iron content but significantly increased levels of serum EPO and Hb in HbE and homozygous NTDT patients (Pootrakul et al., 2003). Taken together, an increase in EPO production in thalassemia patients *per se* will enhance erythropoietic activity to compensate for ineffective erythropoiesis and anemia, while EPO supplementation will stimulate intramedullary erythropoiesis in the bone marrow, decrease extramedullary erythropoiesis in the liver and spleen as well as accelerate the utilization of tissue iron. Conversely, our study has revealed that treatments of GTE, DFP and a combined treatment involving GTE and DFP remarkably decreased levels of plasma EPO, ERFE and Ft, as well as kidney *Epo* mRNA and spleen *Erfe* mRNA in iron-loaded BKO mice when compared with mice who had been given the DI treatment; nevertheless, the combined treatment did not contribute to the efficacy of all of the treatments. Accordingly, all treatments increased the utilization of tissue iron along with the EPO that persisted in the plasma compartment for erythropoiesis. Subsequently, the treatments restored the upregulation of *Epo* and *Erfe* genes as well as increased EPO and ERFE production. Notably, we found that levels of plasma EPO and *Epo* mRNA in the kidneys of iron−loaded BKO mice were several−folds higher than those in WT mice (data not shown). Consistently, subcutaneous injections of recombinant EPO, alone or together with the oral administration of DFP, are recommended for β−thalassemia HbE patients in order to increase iron mobilization from the tissue for erythropoiesis (Cermak, 2006).

ERFE is a glycoprotein hormone, which along another the erythropoietic regulator, is secreted by splenocytes and erythroblasts in response to ineffective erythropoiesis, anemia, hemorrhage, and EPO stimulation leading to the suppression of hepcidin production in hepatocytes. Recently, Kautz and colleagues have reported that the ablation of ERFE moderately improved ineffective erythropoiesis but did not influence the anemic status of the subjects (Kautz et al., 2015). At present, certain drugs or agents, including apo−transferrin, ferroportin inhibitors (e.g., VIT−2763), hepcidin mimetic (e.g., Rusferticle or PTG−300), pyruvate kinase activators (e.g., Mitapivat) and ERFE antibodies, are being researched and developed in order to relieve ineffective erythropoiesis and improve anemia by targeting abnormal iron metabolism in thalassemia patients (Langer and Esrick, 2021). We have previously demonstrated that hepatic *Hamp1* mRNA suppression is associated with a down−regulation of atonal homolog 8, which is a positive regulator of hepcidin transcription that links erythropoiesis with iron−responsive molecules in response to iron−loading anemias (e.g., thalassemia) (Upanan et al., 2015). Inversely, the *Hamp1* expression was significantly up−regulated in the BKO mice treated with a combination of GTE and DFP (Upanan et al., 2017). Interestingly, Kim and colleagues showed that EGCG treatment induced expression and its production of small heterodimer partner-interacting leucine zipper protein in hepatocytes, subsequently turning on bone morphogenetic protein 6−mediated SMAD1/5/8 transactivation of the hepcidin gene, and consequently increasing the hepcidin secretion from the hepatocytes (Kim et al., 2021). Herein, we have revealed that the levels of plasma ERFE and splenic *Erfe* mRNA were considerably higher in iron loaded−BKO mice than in WT mice in compensation for the anemic condition (data not shown). In a similar fashion, plasma ERFE and Ft levels were decreased by either GTE or DFP monotherapy, along with the combination therapy, when compared with mice that had received the DI treatment. Consistently, *Erfe* mRNA levels in the splenic tissue of iron−loaded BKO mice were significantly reduced by all three treatments when compared with mice that had received the DI treatment. Accordingly, treatments of GTE, DFP or a combination of the two successfully resulted in decreases in *Epo* mRNA and EPO production and subsequently decreased *Erfe* mRNA and ERFE production. Furthermore, this increased the *Nrf2* gene expression during incidences of stress erythropoiesis (Guo et al., 2017). In contrast, a recent study has elucidated that hepcidin expression in the livers of mice remained unchanged, although the levels of plasma EPO and ERFE were found to be very high (Altamura et al., 2020). As expected, the decreased ERFE secretion would allow for upregulation in hepcidin gene expression and an increase in hepcidin secretion from hepatocytes, while subsequently decreasing iron absorption from the duodenum and iron efflux from reticuloendothelial macrophages and potentially reducing iron loading in the body.

Patients with secondary iron overload, such as thalassemia patients, are known to suffer from oxidative damage in iron−overloaded tissues, particularly in the liver and spleen (Siegelman et al., 1996; Brissot et al., 2019). DFP is an orally active bidentate iron chelator that has been used for the treatment of iron overload diseases such as thalassemia and neurological disorders; however, the drug can cause agranulocytosis, gastrointestinal disorders, elevated liver enzymes and zinc deficiency. Interestingly, GTE which is most abundant with EGCG, wherein the galloyl and diol−positioned dihydroxyl groups can bind and chelate the iron. Mechanistically, one molecule of either EGCG or EGC is capable of binding and reducing up to four iron (III) atoms (metal:ligand = 2:1) when compared with DFP (metal:ligand = 1:3). In combination, DFP would act as a shuttle to mobilize intracellular iron and transfer the chelated iron to EGCG, which can be accomplished in a sink outside the cells as was consistent with the outcomes of previous studies (Evans et al., 2010; Vlachodimitropoulou Koumoutsea et al., 2015). With regard to iron overload status, we have determined iron accumulation in the liver, spleen and kidney tissues of iron−loaded BKO mice using ferrozine and Perl’s staining methods. Levels of tissue iron were tentatively decreased in mice receiving monotherapy involving GTE and DFP, and in those receiving a combination of the two treatments; nevertheless, the combination therapy was not determined to be significantly more effective than monotherapy. In comparison, using the semi−quantitative Perl’s staining histochemical method, iron distribution in these three tissues were decreased significantly in mice receiving all treatments.

In addition, lipid−peroxidation products (such as TBARS) were measured in those three tissues and plasma samples. We have demonstrated that tissue TBARS levels tended to be decreased in mice receiving all three treatments, while plasma TBARS levels decreased significantly in all cases in which the GTE monotherapy and the combined therapy were determined to be more effective than DFP. According to the relevant iron−chelating property, GTE; particularly EGCG *per se*, uses diol−phenolic groups to chelate Fenton iron and acts as a sink to shuttle the chelated iron from DFP. Surprisingly, quercetin, which is a polyphenolic antioxidant, has been reported to function as a shuttle for the mobilization of intracellular labile iron to plasma transferrin and as a suppressor of iron pro−oxidant activity (Baccan et al., 2012). Similarly, EGCG and DFP monotherapy, and a combination of the two, behave like this to counteract the chain−reaction lipid peroxidation in the cellular and plasma compartment, wherein the combination treatment was observed to be more effective in the plasma than monotherapy. In addition, Bao and colleagues have revealed that EGCG was able to bind a redox iron and protect against iron-induced oxidative renal injuries (Bao et al., 2013). Importantly, GTE and DFP monotherapy, and a combination of the two, reduced plasma NTBI levels and tissue iron deposition, of which the combination was observed to be more effective. Remarkably, it also restored the decreased hepatic *Hamp1* mRNA expression levels in ferrocene−fed BKO mice (Upanan et al., 2015). Consistently, we have revealed that GTE effectively chelated the iron in the liver and decreased the ROS in ferrocene−fed BKO mice (Srichairatanakool et al., 2006; Saewong et al., 2010; Koonyosying et al., 2020a). Lastly, we have confirmed that levels of TBARS are used as simple biomarkers for the assessment of oxidative status in β−thalassemia patients (Meral et al., 2000; Ondei Lde et al., 2013) in the plasma, liver, spleen and kidneys of iron dextran−loaded BKO mice were dramatically decreased after treatments of GTE, DFP and a combination of the two when compared to mice that had not received treatment (DI group). This was consistent with the outcomes of our previous studies (Thephinlap et al., 2007; Ounjaijean et al., 2008; Saewong et al., 2010; Koonyosying et al., 2018). In terms of limitations, some BKO mice died during the loading of iron dextran, and only a few iron−overloaded BKO mice remained in the experiments. A small volume of heart blood was obtained from the mice but was insufficient to conduct other erythropoietic regulator experiments.

With regard to the toxicity of green tea, an oral administration of 2 g EGCG/kg BW/d for 10 days, along with an intravenous injection of 50 mg EGCG/kg/d, did not induce genotoxicity in murine models (Isbrucker et al., 2006). However, multiple once-daily treatments of 500 and 750 mg EGCG/kg BW decreased the survival rate of mice by 20 and 75%, respectively, while a dose of 750 mg EGCG/kg increased the levels of serum alanine transferase (ALT) activity indicating hepatotoxicity (Lambert et al., 2010). Consistently, the European Food Safety Agency has documented that signs of liver damage may be indicated in humans who consume green tea at a dose of up to 800 mg EGCG/d but are not found for doses below 800 mg EGCG/d. Likewise, healthy adults who consumed GTE containing 323.6 mg EGCG for 12 weeks demonstrated improved visual function and did not exhibit any adverse effects (Maeda-Yamamoto et al., 2018). Moreover, Ullman and colleagues have demonstrated that healthy subjects tolerated a single intake of EGCG up to 1600 mg (approximately 25 mg/kg), showing maximum plasma concentration of EGCG in a range of 130–3392 ng/ml and half-life values between 1.9 and 4.6 h (Ullmann et al., 2003). Furthermore, a comprehensive systemic review by the United States Pharmacopeia has reported that hepatotoxicity occurred as a result of an increase in the intake of EGCG from 140 mg to approximately 1,000 mg/day (Sarma et al., 2008; Oketch-Rabah et al., 2020). We have previously revealed that the oral administration of 50 mg EGCG equivalent/kg/day did not influence the serum levels of ALT and aspartate transferase (AST) activity in wild−type (WT) and iron-loaded beta−thalassemic (BKO) mice. (Koonyosying et al., 2018). Additionally, we determined that decreases in blood urea nitrogen and serum redox iron levels did not cause any adverse effects in β−thalassemia patients who had consumed a GTE (35.5 mg EGCG equivalent)−curcumin drink for 60 days (Koonyosying et al., 2020b). In the present study, we gavage fed a GTE solution of 50 mg EGCG equivalent/kg BW/d to mice, which was equal to 1.25 mg EGCG each/d if the mice weighed approximately 25 g on mice for 60 days. This led to a cumulative amount of 75 mg of EGCG. Hence, we increased the GTE dose up to 50 mg EGCG equivalent/kg/d as a therapeutic approach against iron-overloaded oxidative stress conditions in β−thalassemia mice, while the maximal dose of 25 mg EGCG/kg/d was used in healthy humans.

Hepatic siderosis can result from iron overload conditions including hereditary hemochromatosis, secondary hemosiderosis (such as thalassemia), alcohol-related liver disease, non-hereditary hemochromatosis, ferroportin disease and multiple blood transfusions. Consequently, the accumulation of iron is directly toxic to hepatocytes, which act as a Fenton catalyst for ROS generation and a stimulus for collagen production. In the present study, we used Perl’s staining method for histochemical detection of ferric iron deposits and the H & E staining method for histopathological examination of changes in cell morphology in the liver, spleen and kidneys. Furthermore, our findings have revealed monotherapy involving GTE, DFP and a combination therapy were effective in treating BKO mice with iron overload and were nontoxic to the animals. Consistently, our previous studies have indicated that the oral administration of GTE (50 mg EGCG equivalent/day) was acceptable for the iron−loaded BKO mice (Upanan et al., 2015; Koonyosying et al., 2018). We believe that an equivalent dose of 50 mg EGCG was effective enough to mobilize excessive iron in tissues and plasma compartments but would not interfere with functional iron levels in cells under iron overload conditions.

## 5 Conclusion

Treatments of iron-loaded BKO mice with GTE and DFP did not change any of the RBC parameters but considerably decreased the plasma levels of EPO, ERFE, ferritin and TBARS. Surprisingly, the treatments did lower the levels of *Epo* and *Erfe* gene expression, iron deposition and TBARS in the liver, spleen, and kidneys. However, the combination therapy was not observed to be more effective than monotherapy in reducing the levels of these parameters with the exception of the plasma TBARS. This study has reported on the nutraceutical effects of green tea extract, erythropoietic regulators, stress erythropoiesis, the iron mobilization of relevant tissues and the amelioration of oxidative organ damage. In the future, EGCG−rich GTE will require encapsulating in nano−carriers (e.g., alginate, chitosan, sodium tripolyphosphate or polyethylene glycol) in order to enhance the quality, efficiency and health−promoting effects of this substance at minimal doses. Moreover, the efficacy of the nano−encapsulated green tea on stress erythropoiesis should be clinically investigated in β−thalassemic patients, particularly in non−transfusion−dependent thalassemia patients who have exhibited increased ferrokinetics.

## Data Availability

The original contributions presented in the study are included in the article/Supplementary Materials, further inquiries can be directed to the corresponding authors.

## Glossary

ANOVA: one-way analysis of variance
BHT: butylated hydroxytoluene
BKO: beta-globin knockout
C: catechin
cDNA: complementary deoxyribonucleic acid
DEPC: diethylpyrocarbonate
DFO: desferrioxamine
DFP: deferiprone
DFX: deferasirox
DI: deionized water
ECG: epicatechin-3-gallate
EGC: epigallocatechin
EGCG: epigallocatechin-3-gallate
ELISA: enzyme-linked immunosorbent assay
EPO: erythropoietin
Epo: erythropoietin gene
ERFE: erythroferrone
Erfe: erythroferrone gene
ETOH: ethanol
G6PD: glucose-6−phosphate dehydrogenase
GSH: glutathione
GSSG: oxidized glutathione
GTE: green tea extract
Hb: hemoglobin
Hct: hematocrit
Hprt: hypoxanthine-phosphoribosyltransferase gene
H & E: hematoxylin and eosin
IE: ineffective erythropoiesis
MCH: mean corpuscular hemoglobin
MCHC: mean corpuscular hemoglobin concentration
MCV: mean corpuscular volume
NS: nonsignificant
NSS: normal saline
NTBI: non−transferrin bound iron
NTDT: non−transfusion-dependent thalassemia
OD: optical density
PBS: phosphate buffered saline
PCR: polymerase chain reaction
qRT−PCR: quantitative reverse-transcription polymerase chain reaction
RBC: red blood cells
RDW: red blood cell distribution width
RNA: ribonucleic acid
RP−HPLC: reverse-phase high performance liquid chromatography
ROS: reactive oxygen species
SEM: standard error of the mean
TBA: thiobarbituric acid
TBARS: thiobarbituric acid-reactive substances
TCA: trichloroacetic acid
TDT: transfusion-dependent thalassemia
TGA: thioglycolic acid
TMP: tetramethylpyrazine
UV−VIS: ultraviolet-visible
WI: weight index
WT: wild type
